# The subcommissural organ of the rat secretes Reissner's fiber glycoproteins and CSF-soluble proteins reaching the internal and external CSF compartments

**DOI:** 10.1186/1743-8454-5-3

**Published:** 2008-01-24

**Authors:** Karin Vio, Sara Rodríguez, Carlos R Yulis, Cristian Oliver, Esteban M Rodríguez

**Affiliations:** 1 Instituto de Anatomía, Histología y Patología, Facultad de Medicina, Universidad Austral de Chile, Valdivia, Chile

## Abstract

**Background:**

The subcommissural organ (SCO) is a highly conserved brain gland present throughout the vertebrate phylum; it secretes glycoproteins into the cerebrospinal fluid (CSF), where they aggregate to form Reissner's fiber (RF). SCO-spondin is the major constituent protein of RF. Evidence exists that the SCO also secretes proteins that remain soluble in the CSF. The aims of the present investigation were: (i) to identify and partially characterize the SCO-secretory compounds present in the SCO gland itself and in the RF of the Sprague-Dawley rat and non-hydrocephalic hyh mouse, and in the CSF of rat; (ii) to make a comparative analysis of the proteins present in these three compartments; (iii) to identify the proteins secreted by the SCO into the CSF at different developmental periods.

**Methods:**

The proteins of the SCO secreted into the CSF were studied (i) by injecting specific antibodies into ventricular CSF *in vivo*; (ii) by immunoblots of SCO, RF and CSF samples, using specific antibodies against the SCO secretory proteins (AFRU and anti-P15). In addition, the glycosylated nature of SCO-compounds was analysed by concanavalin A and wheat germ agglutinin binding. To analyse RF-glycoproteins, RF was extracted from the central canal of juvenile rats and mice; to investigate the CSF-soluble proteins secreted by the SCO, CSF samples were collected from the cisterna magna of rats at different stages of development (from E18 to PN30).

**Results:**

Five glycoproteins were identified in the rat SCO with apparent molecular weights of 630, 450, 390, 320 and 200 kDa. With the exception of the 200-kDa compound, all other compounds present in the rat SCO were also present in the mouse SCO. The 630 and 390 kDa compounds of the rat SCO have affinity for concanavalin A but not for wheat germ agglutinin, suggesting that they correspond to precursor forms. Four of the AFRU-immunoreactive compounds present in the SCO (630, 450, 390, 320 kDa) were absent from the RF and CSF. These may be precursor and/or partially processed forms. Two other compounds (200, 63 kDa) were present in SCO, RF and CSF and may be processed forms. The presence of these proteins in both, RF and CSF suggests a steady-state RF/CSF equilibrium for these compounds. Eight AFRU-immunoreactive bands were consistently found in CSF samples from rats at E18, E20 and PN1. Only four of these compounds were detected in the cisternal CSF of PN30 rats. The 200 kDa compound appears to be a key compound in rats since it was consistently found in all samples of SCO, RF and embryonic and juvenile CSF.

**Conclusion:**

It is concluded that (i) during the late embryonic life, the rat SCO secretes compounds that remain soluble in the CSF and reach the subarachnoid space; (ii) during postnatal life, there is a reduction in the number and concentration of CSF-soluble proteins secreted by the SCO. The molecular structure and functional significance of these proteins remain to be elucidated. The possibility they are involved in brain development has been discussed.

## Background

The subcommissural organ (SCO) differentiates at an early stage of ontogenetic development in all vertebrates [1] and, with the exception of a few species (anthropoids and bats) [2], it remains fully active throughout life. The secretion of this gland is released into the ventricular cerebrospinal fluid (CSF) where most of it condenses to form a filamentous structure named after Reissner [3] as Reissner's fiber (RF). RF is formed by the assembly of complex-type, high molecular weight glycoproteins secreted by the SCO into the cerebral aqueduct; it is a dynamic structure that continuously grows caudally by the addition of newly released molecules to its cephalic end [2,4]; it extends throughout the aqueduct of Sylvius, fourth ventricle and central canal of the spinal cord [4,5]. When arriving at the terminal ventricle of the central canal at the filum, RF glycoproteins undergo chemical modification, disassembly and passage into neighbouring vessels [6,7]. RF has the capacity to bind and transport away compounds such as dopamine, L-DOPA and serotonin, thus contributing to the clearance of these compounds from the CSF [8,9].

The identification and characterization of the secretory compounds of the SCO have been the subject of numerous investigations and have contributed to partial clarification of its function. Immunoblot analyses of bovine SCO using antibodies against RF glycoproteins have led to the identification of high molecular weight glycoproteins of 540, 450, 320 and 190 kDa. Evidence has been obtained indicating that the 540 and the 320 kDa compounds would correspond to precursor forms [10-12]. In the SCO of chick embryos, del Brio *et al*. have determined the presence of three compounds of 540, 320 and 230 kDa [13]. In the SCO of the elasmobranch *Scyliorhinus canicula*, five compounds of 600, 475, 400, 145 and 35 kDa have been identified [14]. Antibodies raised against the precursor form of 540 kDa and the processed form of 450 kDa synthesized by the bovine SCO, when used to immunostain the bovine and rat SCO, react with the bovine SCO but not with the rat SCO. At variance, the antibody against the bovine SCO 320 kDa band reacts with both, bovine and rat SCO [11,12]. This is the only information available concerning the secretory compounds of the SCO of murine species.

Ontogenetic studies have revealed that the SCO starts to express a secretory activity much earlier than the appearance of the first RF [1,15]. In the rat, the SCO is well developed and immunoreactive with the anti-RF antibodies at embryonic day 14 (E14). However, aggregated secretory material and a RF proper first appear during the first postnatal week [1]. These findings suggest that the embryonic SCO secretes compounds that remain soluble in the CSF, thus differing from RF proteins, which aggregate. The existence of CSF-soluble compounds secreted by the SCO gained support by immunochemical studies in human and rabbit CSF [2,16]. Recently, Hoyo-Becerra *et al*. [17] have described the presence in the CSF from chick embryos of proteins that are reactive with anti-RF antiserum (AFRU). Efforts to detect SCO glycoproteins in the bovine CSF have failed [18,19]. None of previous studies have dealt with the characterization of the SCO secretory glycoproteins present in the gland itself, in the CSF and in the RF of the same animal species and using immunoblot methodology; this has prevented a comparative analysis of the secretory proteins before they are released and after they either assemble into RF or remain soluble in the CSF. Thus, whether the SCO proteins forming RF and the SCO proteins solubilized in the CSF are the same, or similar or unrelated compounds has yet to be determined.

The primary structure of the major constituent of bovine RF, SCO-spondin, has been fully established as a large N-glycosylated protein (450 kDa) [20,21]. Several lines of evidence indicate that SCO-spondin plays a role in CNS development. SCO-spondin belongs to a protein superfamily exhibiting conserved motifs of the thrombospondin type 1 repeat [21]. Proteins of this family are strongly expressed during mammalian CNS development and are involved in mechanisms of cellular adhesion and axonal pathfinding [22]. It has not yet been established whether SCO-spondin itself or its processed products are present in and circulate through the CSF compartments. Neither has it been established whether SCO-spondin or related compounds are actually secreted into the embryonic CSF. This information is essential when interpreting the potential role of SCO-spondin in CNS development.

The present investigation was designed to investigate the rat and mouse SCO glycoproteins in order (i) to identify and partially characterize the secretory compounds present in the gland itself, in the RF and in the CSF (rat only); (ii) to make a comparative analysis of the proteins present in these three compartments, in order to establish to what extent they share gel migration, immunoreactive and lectin-binding properties; and (iii) to identify the proteins secreted by the SCO into the CSF at different developmental periods. The identification of the CSF-soluble secretion of the SCO during embryonic and juvenile life may throw some light on the function of this gland. Indeed, such CSF-soluble proteins would circulate throughout both the internal and external CSF compartments and could reach distant targets. Furthermore, differences in the type or concentration of these CSF-soluble proteins at different developmental stages may open new avenues for SCO research.

## Methods

### Animals

Sprague-Dawley rats and *hyh *mice: Sprague-Dawley rats and *hyh *mice were obtained from The Jackson Laboratory (Bar Harbor, ME, USA) and bred in two colonies (Facultad de Medicina, Universidad Austral de Chile, Valdivia, Chile). Housing, handling, care and processing of animals were carried out according to the regulations approved by the council of the American Physiological Society. A local university committee has approved the experimental protocol. Animals were fed *ad libitum *with rodent food and maintained under a constant 12-h light/dark photoperiod and at a constant temperature. Female rats were checked for the presence of a vaginal plug after overnight mating with a male. At days 18, 19 and 20 of pregnancy, pregnant rats were weighed and anesthetized with an intraperitoneal injection of ketamine (40 mg/kg) and acepromazine (100 mg/kg); embryos were removed and CSF was collected from the cisterna magna.

For mice, the *hyh *mutation arose spontaneously on the C57BL/10J. They were then outcrossed onto a B6C3Fe-a/a background (C57BL/6J × C3HeB/FeJ-a/a). Hence, the genetic background of *hyh *mice strain (B6C3Fe a/a-Napahyh/J) is C57BL/6J × C3HeB/FeJ-a/a. In the present study only non-hydrocephalic mice were used. No differences between wild type and heterozygous mice were detected with the methods used.

### Immunocytochemistry

The brains of 10 Sprague-Dawley rats, PN2, were fixed by intravascular perfusion with Bouin fixative and embedded in paraffin. Sections were processed by the immunoperoxidase method of Sternberger *et al*. [23]. The following primary antibodies were used: (i) An antiserum developed in rabbits against bovine RF-glycoproteins extracted in a medium containing urea (AFRU, A = antiserum, FR = Fiber of Reissner, U = urea; [24]), (ii) An antiserum developed in rabbits against a synthetic 15-aminoacid peptide with a sequence deduced from a region of the SCO-spondin protein that does not correspond to any of the repeats present in this molecule (anti-P15; [21,25]). This peptide sequence has no homology with any of the proteins recorded in the Gene Bank; hence anti-P15 can be regarded as a specific antibody for SCO-spondin [25]. Sections were sequentially incubated in: (i) AFRU, dilution 1:1000, or anti-P15, dilution 1:500, for 18 h; (ii) secondary antibody (anti-rabbit IgG, raised in our laboratory), diluted 1:15, for 30 min; (iii) rabbit PAP (Dako, Carpinteria, CA, USA), diluted 1:75, for 30 min. All immunocytochemical reactions were visualized by the histochemical detection of peroxidase using hydrogen peroxide (Merck, Darmstadt, Germany) and 3-3'diaminobenzidine tetrahydrochloride (DAB, Sigma, Madrid, Spain). Omission of the incubation in the primary antibody was used as a control.

### Injection of antibodies into the rat CSF

Eight PN2 Sprague-Dawley pups were anesthetized with an intraperitoneal injection of ketamine (40 mg/kg) and acepromazine (100 mg/kg); anesthesia lasted throughout the experiment. The head of the pup was immobilized by placing it into a paraffin cast specially adapted to the head of PN2 rats. Using a dissecting microscope, a 27-gauge cannula connected to a perfusion pump, was inserted into the left lateral ventricle; 3 μl of undiluted AFRU (*n *= 4) or anti-P15 (*n *= 4) sera were infused over 5 min. Two hours after antibody administration, the brain was dissected out and fixed by immersion in Bouin's fixative for 2 d. After dehydration in alcohols, the brains were embedded in paraffin. For the demonstration of binding sites of the injected antibodies, sections were sequentially incubated with anti-rabbit IgG, diluted 1:15, for 30 min, and rabbit PAP (Dako, Carpinteria, CA, USA), diluted 1:75, for 30 min. This was followed by the diaminobenzidine reaction.

### Reissner's fiber extracts

Bovine spinal cord from adult cows was obtained as described previously [24] and RF was collected by perfusing the central canal of the spinal cord with saline. RF of 10 juvenile rats (PN30) and 8 juvenile mice (PN30) was collected in the following way: after euthanasia with ketamine, the spinal cord was dissected out, immersed in buffered saline and divided into several segments by making transverse cuts with a razor blade. Under a dissecting microscope, a cylinder of the grey matter surrounding the central canal was obtained by doing four longitudinal cuts; this block of tissue contained the central canal and the corresponding stretch of RF. The bovine and murine RFs were extracted in 50 mM ammonium bicarbonate, pH 7,4, containing 0.5 mM phenyl-methylsulfonyl fluoride (PMSF). The protein concentration of bovine RF extract [26] was 0.8 μg/μl, rat central canal/RF extracts ranged from 8.0 – 8.6 μg/μl and mouse central canal/RF extracts ranged from 5.0 – 6.4 μg/μl. Samples were stored at -70°C until used.

### SCO extracts

Fifty PN30 rats and thirty PN30 mice were used. The animals were sacrificed under ketamine anesthesia, the brain was dissected out and a block of tissue containing the SCO and the posterior commissure was obtained under a dissecting microscope; post-mortem interval was 2–5 min. Six SCOs were pooled to make one sample. Each sample was extracted in 300 μl of 50 mM ammonium bicarbonate, pH 7,5, and 0,5 mM phenyl-methylsulfonyl fluoride (PMSF), homogenized, sonicated in ice for 10 s and centrifuged at 12.000 g, for 45 min, at 4°C. Three aliquots of 100 μl, each containing the equivalent of two SCOs, were obtained from each extracted sample. Protein content of rat SCO extracts ranged from 1.5 to 2.2 μg/μl and of mouse SCO extracts from 1.2 – 1.3 μg/μl. Extracts were stored at -70°C. The bovine SCO of adult animals was collected and extracted (protein concentration 1.2 – 1.35 μg/μl) as previously reported [12].

### Cerebrospinal fluid collection

Rats: E18 (*n *= 18), E19 (*n *= 22) and E20 (*n *= 20) rat embryos were used for CSF collection. CSF samples were obtained through a 30-gauge needle inserted into the cisterna magna. About 10–15 μl were obtained from each embryo. Pups at PN1 (*n *= 12) and PN7 (*n *= 20) were anesthetized with ketamine (40 mg/kg) and acepromazine (100 mg/kg), the head flexed and a 27-gauge needle inserted into the cisterna magna. In PN30 rats (*n *= 22) CSF was collected from the cisterna magna according to Rodríguez *et al*. [9]. Occasionally, severence of a blood vessel caused contamination of the samples with blood and these samples were discarded. About 25–50 μl of CSF were obtained from each pup and up to 100 μl from each PN30 rat. CSF samples were collected into Eppendorf tubes and centrifuged twice to remove cells or cell debris. Average protein concentrations of E18, E20, PN1, PN7 and PN30 CSF samples were: 2.4, 1.8, 1.5, 1.0 and 0.4 μg/μl, respectively. Samples were stored at -70°C.

### Immunoblot analyses

The following samples were used for blotting: (i) 50 μl samples of SCO extracts, containing the equivalent of 0.02 bovine SCO, 1 mouse SCO and 1 rat SCO; (ii) 50 μl samples of RF extracts containing the equivalent of 0.001 bovine RF, 0.1 mouse RF and 0.1 rat RF; (iii) 15 μl of undiluted/non concentrated CSF from embryonic and postnatal rats. Samples were subjected to SDS-PAGE using a 5–15% polyacrylamide linear gradient. Proteins were transferred to nitrocellulose membranes [27]; to block non-specific binding, blots were saturated with 5% non-fat milk in 0.1 M PBS containing 0.15 mM NaCl and 0.1% Tween-20 (Sigma, Madrid, Spain), for 90 min. Two primary antisera were used for immunoreaction of blots of SCO and RF: (i) AFRU, 1:25,000 dilution, for 2 h; (ii) anti-P15 [24], 1:1,000 dilution, for 2 h. Anti-rabbit IgG-HRP (Pierce, Rockford, IL, USA) was used at 1:25,000 dilution, for 1.5 h. For immunoblotting of CSF samples, the following protocol was used: (i) AFRU, at 1:5,000 dilution and anti-rabbit IgG-HRP at 1:25,000 dilution, for 2 h; (ii) anti-P15, at 1:1,000 dilution and anti-rabbit IgG-HRP at 1:60,000 dilution, for 2 h. Incubations were at room temperature and in darkness. Immunoreactive polypeptides were detected by using an enhanced chemiluminescence (ECL) system (Super Signal, Pierce, Rockford, IL, USA) as instructed by the manufacturer. Molecular weight standards in the range of 10–250 kDa were used (Bio-Rad, Hercules, CA, USA). Control blots were processed as above without the primary antibody. Immunoblotting of samples was repeated as follows: SCO of rat and mouse × 10; RF of rat and mouse × 5; rat CSF of E18 and E20 embryos × 5; rat CSF collected at PN1, PN7 and PN30 × 10. Immunoblots were digitized (*n *= 4 for each condition analysed) and linear densitograms were obtained using the UN – SCAN-IT software (Silk Scientific, Orem, UT, USA). Statistical analyses were performed using the Prism software (GraphPad Software, San Diego, CA, USA) applying the 1-way Anova and Tukey's test.

### Lectin binding

Blots of bovine, rat and mouse SCO in parallel to those used for immunoblotting, were used to test for lectin binding. Blots were sequentially incubated with (i) oxidized bovine serum albumin (BSA, Winkler, Santiago, Chile), (ii) concanavalin A (Con A; affinity = mannose, glucose; Sigma, St. Louis, MO, USA), 0.2 μg/ml for 1 h; (iii) anti-Con A developed in rabbits (Sigma, St. Louis, MO, USA), 1:10,000 dilution for 1 h at room temperature; (iii) anti-rabbit IgG-HRP (Pierce, Rockford, IL, USA) 1:25,000, for 1.5 h; (iv) binding was detected by using an enhanced chemiluminescence system (see above). The same protocol was used for binding of wheat germ agglutinin (WGA; affinity = glucosamine, sialic acid, Sigma, St. Louis, MO, USA). WGA was used at a concentration of 0.5 μg/ml and anti-WGA (Sigma, St. Louis, MO, USA) was used at a 1:5,000 dilution.

Con A has affinity for internal, and preferentially for terminal, mannose residues; this makes it a good marker for newly synthesised glycoproteins that have not yet been processed through the Golgi apparatus (precursor forms). At variance, WGA has affinity for two sugar residues (glucosamine, sialic acid) added to the glycoprotein by the Golgi apparatus, thus being a good marker of post-Golgi compounds (processed forms) [28,29].

## Results

### Immunocytochemistry

**Figure 1:**
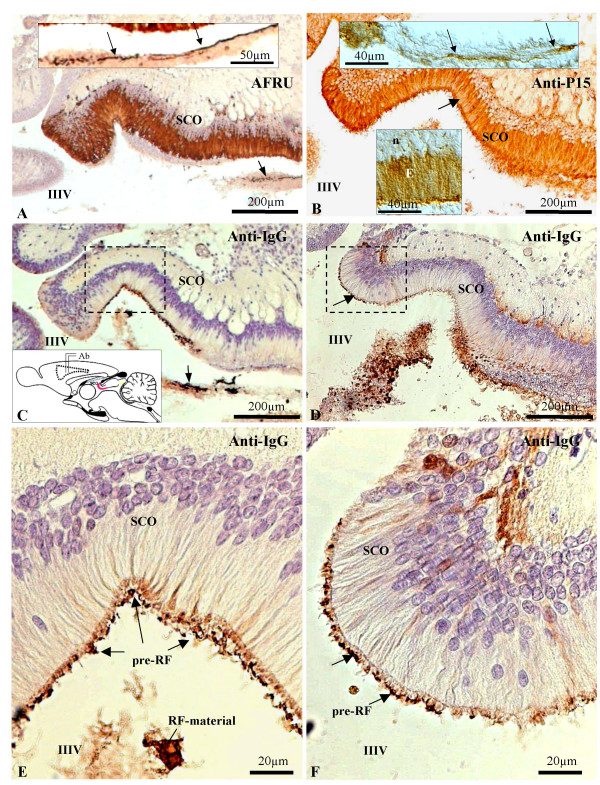
A, C, E: Paraffin sagittal sections through the subcommissural organ (SCO) of a PN2 rat after a single injection of AFRU into a lateral ventricle (inset 1C, labeled Ab). A: Immunostaining using AFRU (raised in rabbits) as primary antibody. The SCO is immunoreactive. Arrow points to RF-material. Insert: Detailed magnification of RF fibrils immunoreactive with AFRU (arrows). C: Adjacent section to that shown in A, immunostained using anti-rabbit IgG as primary antibody to reveal the antibody (AFRU) administered *in vivo*. The latter appears exclusively bound to the newly released secretory material aggregated on the surface of the SCO to form the pre-RF (square) and to aggregated RF-material lying in the ventricle (arrow). E: Detailed magnification of area framed in C showing the location of the antigen-antibody complexes formed *in vivo *at the pre-RF layer on the SCO surface and at the aggregated RF-material. B, D, F: Paraffin sagittal sections through the SCO of a PN2 rat after a single injection of anti-P15 into a lateral ventricle. B: Sagittal section through the SCO immunostained with anti-P15. The ependymal cells of the SCO appear immunostained (arrow). Lower insert: detailed magnification of the secretory ependyma (E) showing the location of the immunoreactive material in the supranuclear cytoplasm. n: nuclei. Upper insert: detailed magnification of RF fibrils immunoreactive with anti-P15 (arrows). D: Section immunostained with anti-rabbit IgG as primary antibody to reveal the location of the antibody administered *in vivo *at pre-RF (arrow). F: Detailed magnification of area framed in previous figure showing the location of the antigen-antibody complexes formed *in vivo *at the pre-RF (arrows). IIIV: third ventricle. Most sections counterstained with hematoxilin.

**Figure 2:**
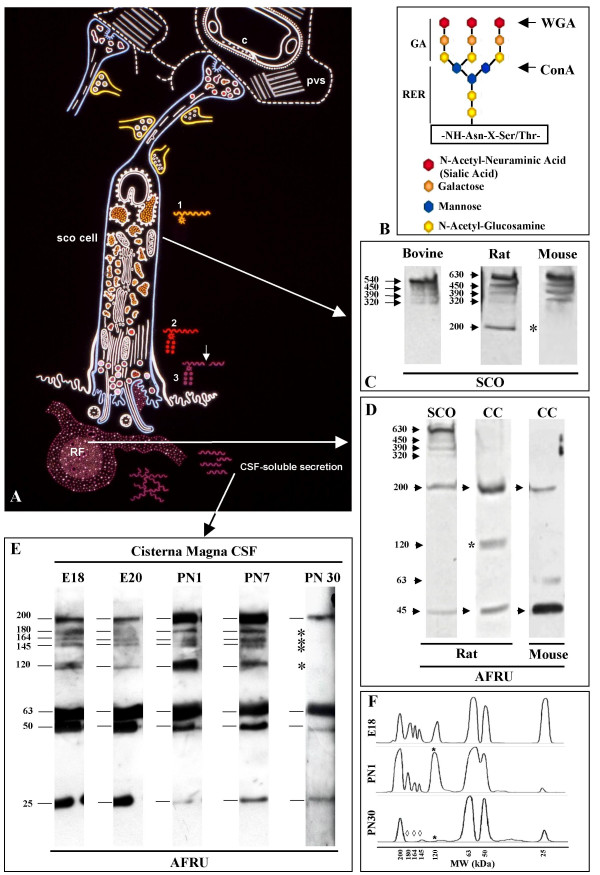
A: Schematic representation of a secretory ependymal cell of the rat SCO. In the rough endoplasmic reticulum secretory proteins have N-linked, high mannose type oligosaccharides (1). In the Golgi cisternae glucosamine, galactose and sialic acid are conjugated to the saccharide core (2). In the secretory granules and in the released secretion, the backbone protein of the secretory glycoproteins undergoes cleavage (3, arrow) [12, 28]. The secretory glycoproteins released into the ventricle may become densely packed forming Reissner's fiber (RF) or may remains soluble in the cerebrospinal fluid (from Rodriguez *et al*. [16]). B: Schematic representation of a N-linked, complex type, oligosaccharide. The core of glucosamine and mannose are conjugated in the rough endoplasmic reticulum (RER); glucosamine, galactose and sialic acid are conjugated in the Golgi apparatus (GA). Concanavalin A (Con A) has affinity for internal and terminal mannose residues; wheat germ agglutinin (WGA) has affinity for terminal residues of glucosamine and sialic acid. C: Immunoblotting analysis of the subcommissural organ (SCO) using AFRU as primary antibody. Four immunoreactive polypeptides are detected in the adult bovine SCO, five in the PN30 rat SCO and four in the PN30 mouse SCO. Numbers: refer to molecular weight in kDa; arrows: secretory compounds; asterisk indicates the absence of 200 kDa compound. D: Immunoblotting analyses using AFRU as primary antibody of extracts of rat SCO and rat and mouse central canal (CC) at PN30. Numbers refer to molecular weight in kDa. Two polypeptides of 200 and 45 kDa were present in all samples. Asterisk: immunoreactive band of 120 kDa present only in the rat central canal. E: Western blot analysis of cisternal CSF collected from E18, E20, PN1, PN 7 and PN30 rats. Numbers refer to molecular weight in kDa. Eight immunoreactive polypeptides are detected in the CSF collected perinatally (E18, E20, PN1, PN7) (horizontal lines). Four of these compounds are missing from the CSF of PN30 rats (asterisks). F: Densitometric linear scannings of CSF immunoblots at E18, PN1 and PN30. The statistical analysis using four immunoblots each revealed a significant difference in luminescence value of the 120 kDa band between E18 and PN1 (*, *P *< 0.001), and between PN1 and PN30 (*P *< 0.001). Differences between E18 and PN30 in the 180, 164 and 145 kDa bands were also significant (◇, *P *< 0.05).

The compounds secreted by rat SCO were identified using two antibodies. One is directed against bovine RF constitutive proteins (AFRU), whilst anti-P15 is directed against a 15-aminoacid sequence of SCO-spondin, the main constitutive protein of RF. Both antibodies are highly specific for the rat SCO secretion, located either intracellularly or extracellularly in the form of aggregated RF-material (Fig. 1A, B, 2). The SCO-specificity of AFRU and anti-P15 is also evident when used *in vivo*. After the intraventricular administration of these antibodies, they bound exclusively to the SCO secretory material that had been released into the ventricle and that had aggregated into the form of pre-RF or RF [4] (Fig. 1C–F, 2A).

### Analysis of rat and mouse SCOextracts

**Figure 3:**
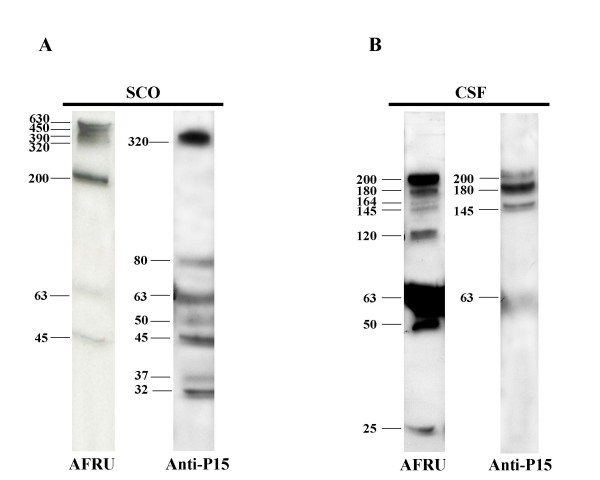
Immunoblots of rat SCO at PN30 (panel A) and cisternal CSF at PN7 (panel B), using AFRU and anti-P15 as primary antibodies. The four AFRU-immunoreactive compounds with the largest molecular mass (630, 450,390, 320 kDa) are present in the SCO and missing from the CSF. Two other compounds (200, 63 kDa) are present in the SCO and CSF (processed forms?). The CSF has four bands (180, 164, 145, 120 kDa) not seen in the SCO. The SCO has only one large molecular mass compound reacting with anti-P15 (specific for SCO-spondin) and several bands of lower molecular masses. The CSF compounds of 200, 180, 145 and 63 kDa, reacting with both antibodies are most likely to be SCO-spondin derivatives.

The secretory compounds of the SCO of PN30 rats and mice were analysed in parallel with the well known secretory glycoproteins of the bovine SCO [11,12]. Despite the small size of the rat and mouse SCO, the use of a high-sensitivity Western blot system based on enhanced chemiluminescence enabled demonstration of the secretory compounds using samples containing the equivalent of one SCO. As reported previously [12], the Western blot analysis of the bovine SCO, using AFRU as primary antibody, revealed polypeptides of 540, 450, and 320 kDa (Fig. 2C). The high sensitivity of the method used in the present study, allowed detection of an additional immunoreactive band of 390 kDa (Fig. 2C). The rat and mouse SCO displayed a pattern of AFRU-immunoreactive bands that differed from that of the bovine SCO (Fig. 2C). The 540-kDa polypeptide present in the bovine SCO was not detected in rat and mouse SCO (Fig. 2C). The rat SCO displayed AFRU-immunoreactive bands of 630, 450, 390, 320 and 200 kDa (Fig. 2C, Tables 1, 2). In a few blots, bands of 63 and 45 kDa were also detected (Figs. 2D, 3A, Tables 2, 3). The compounds of 630 and 200 kDa were absent from the bovine SCO.

**Table 1:** Immunoreactivity to AFRU and lectin binding of proteins extracted from the bovine, rat and mouse subcommissural organ

**MW**	*Bovine SCO (adult)			**Rat SCO (PN30)**			**Mouse SCO (PN30)**		
	**AFRU**	**Con A**	**WGA**	**AFRU**	**Con A**	**WGA**	**AFRU**	**Con A**	**WGA**
**630**				+	+	-	+	+	-
**540**	+	+	-						
**450**	+	+	+	+	+	+	+	+	+
**390**	+	+		+	+	-	+	+	-
**320**	+	+	-	+	+	+	+	+	+
**200**				+	+	+			

MW, molecular weights in kilo Daltons; SCO, subcommissural organ; AFRU, antibody against all glycoproteins of bovine RF; Con A, concanavalin A (affinity = mannose, glucose); WGA, wheat germ agglutinin (affinity = glucosamine, sialic acid); +, positive reaction; -, negative reaction. *From Nualart *et al*. 1991 [12].

**Table 2:** Comparative analysis of AFRU-immunoreactive proteins extracted from the rat subcommissural organ, Reissner's fiber and from the cisterna magna CSF

**MW**	**SCO (PN30)**			**RF (PN30)**	**CSF (E18-PN7)**	**CSF (PN30)**
	**AFRU**	**ConA**	**WGA**	**AFRU**	**AFRU**	**AFRU**
**630**	+	+	-			
**450**	+	+	+			
**390**	+	+	-			
**320**	+	+	+			
**200**	+	+	+	+	+	+
**180**					+	
**164**					+	
**145**					+	
**120**				+	+	
**63**	*			*	+	+
**50**				*	+	+
**45**	*			+		
**25**				*	+	+

MW, molecular weights in kilodaltons; SCO, subcommissural organ; RF, Reissner's fiber; AFRU, antibody against all glycoproteins of bovine RF; Con A, concanavalin A (affinity = mannose, glucose); WGA, wheat germ agglutinin (affinity = glucosamine, sialic acid); +, positive reaction consistently present in all samples; *, positive reaction only found in some samples. Two upper boxes indicate putative precursor forms only present in SCO. Lower box indicates the only compound consistently found in SCO, RF and CSF (processed form).

**Table 3:** Immunoreactivity to AFRU and anti-P15 of proteins extracted from the rat subcommissural organ, Riessner's fiber and cisterna magna CSF

**MW**	**SCO (PN30)**		**RF (PN30)**		**CSF (E18-PN7)**	
	**AFRU**	**Anti-P15**	**AFRU**	**Anti-P15**	**AFRU**	**Anti-P15**
**630**	+					
**450**	+					
**390**	+					
**320**	+	+		+		
**200**	+		+	+	+	+
**180**					+	+
**164**				+	+	
**145**				+	+	+
**120**			+	+	+	
**80**		+		+		
**63**	*	*	*	*	+	*
**50**		*	*		+	
**45**	*	*	+	+		
**37**		+				
**32**		+		+		
**25**			*		+	

MW, molecular weights in kilodaltons; SCO, subcommissural organ; RF, Reissner's fiber; AFRU, antibody against all glycoproteins of bovine RF; anti-P15, antibody specific for SCO-spondin; +, positive reaction consistently present in all samples; *, positive reaction only found in some samples. Horizontal box indicates 200 kDa compound consistently found in all samples. The eight AFRU-immunoreactive compounds detected in embryonic CSF were consistently found in all samples; only three of them react with anti-P15.

With the exception of the 200-kDa compound, all high molecular mass compounds present in the rat SCO were also present in the mouse SCO (Fig. 2C).

### Analysis of central canal/Reissner's fiber extracts

Immunoblotting of a tissue extract that included the central canal of the spinal cord and RF of mice and rats at PN30, revealed AFRU-immunoreactive bands most likely corresponding to RF-glycoproteins. In the rat RF, compounds of 200, 120 and 45 kDa were detected in all blots (Fig. 2D, Tables 2, 3). Compounds of 63, 50 and 25 kDa were detected in 2 or 3 of the 5 blots (Tables 2, 3). In the mouse RF the 200-kDa compound was detected in all blots; compounds of 63 and 45 kDa were present in most blots; a polypeptide of 120 kDa was never detected (Fig. 2D).

### Analysis of CSF samples

In order to identify those compounds secreted by the SCO into the CSF and that, at variance with RF-glycoproteins, remain soluble in the CSF, samples of this fluid were obtained from embryo, early postnatal and juvenile rats and analysed by immunoblotting using AFRU and anti-P15.

CSF samples collected from the cisterna magna during the late embryonic life (E18, E19, E20) and the early postnatal life (PN1, PN7) displayed a very similar pattern of AFRU-immunoreactive bands (Fig. 2E). The latter were of 200, 180, 164, 145, 120, 63, 50 and 25 kDa (Fig. 2E, Table 2, 3). This band pattern was found in all blots. There were, however, some differences of intensity of immunoreaction among bands of samples collected at different ages (E18 to PN7; Fig. 2E, F). The 50 and 25 kDa compounds were more strongly reactive in fetal CSF, whilst the 200 and 120 kDa polypeptides were more reactive in PN1 and PN7 samples (Fig. 2E, F).

A marked difference was observed in the cisternal CSF obtained from PN30 rats; the following bands present in previous stages were absent: 180, 164, 145 (*P *< 0.05) and 120 kDa (*P *< 0.001) and only bands for 200, 63, 50, and 25 kDa were seen (Fig. 2E, F, Table 2, 3).

### Comparative analysis of immunoreactive secretory proteins in the rat SCO, RF and CSF

The high sensitivity of the Western blot system has enabled for the first time within the same species, detection of the AFRU-immunoreactive polypeptides present in the SCO, RF and CSF. This, in turn, has made possible the following comparative analysis: (i) the four AFRU-immunoreactive compounds with the largest molecular mass (630, 450,390, 320 kDa) were present in the SCO and missing from RF and CSF (Table 2, 3). These were possibly precursor or partially processed forms. (ii) Two other compounds (200, 63 kDa) were present in SCO, RF and CSF (Tables 2, 3), possibly processed forms; (iii) three compounds (200, 120, 63 kDa) were present in RF and CSF (Table 2) and possibly in a steady-state equilibrium; (iv) CSF of rat embryos had five bands (180, 164, 145, 50, 25 kDa) not detected in the rat SCO or RF at PN30 (Table 2, compare Figs. 2D and 2E); (v) CSF of PN30 rats had two bands (50, 25 kDa) not detected in the rat SCO and inconsistently detected in RF at PN30 (Table 2). The 200-kDa compound was consistently found in all blots of SCO, RF and CSF from rats (Table 2).

### Comparison of immunoblot analysis of SCO, RF and CSF using AFRU and anti-P15

Blots of rat SCO run in parallel and immunoreacted with AFRU and anti-P15 showed: (i) out of the four compounds of large molecular weight revealed by AFRU, only the 320 kDa reacted with anti-P15 (Fig. 3A, Table 3); (ii) the bands of 63 and 45 kDa reacted with both antibodies (Fig. 3A, Table 3); (iii) bands of 80, 37 and 32 kDa only reacted with anti-P15 (Fig. 3A, Table 3).

Blots of rat RF run in parallel and immunoreacted with AFRU and anti-P15 showed: (i) both antibodies reacted with the 200, 120 and 45 kDa compounds (Table 3); (ii) bands of 320, 164, 145, 80 and 32 kDa only reacted with anti-P15 (Table 3).

Blots of CSF of E18, E20 and PN7 rats run in parallel and immunoreacted with AFRU and anti-P15 showed: (i) out of the eight bands revealed by AFRU (200 to 25 kDa), only the 200, 180, 145 and 63 kDa reacted with anti-P15 (Fig. 3B, Table 3).

### Evidence for the existence of two precursor forms of the secretory proteins in the rat and mouse SCO

**Figure 4:**
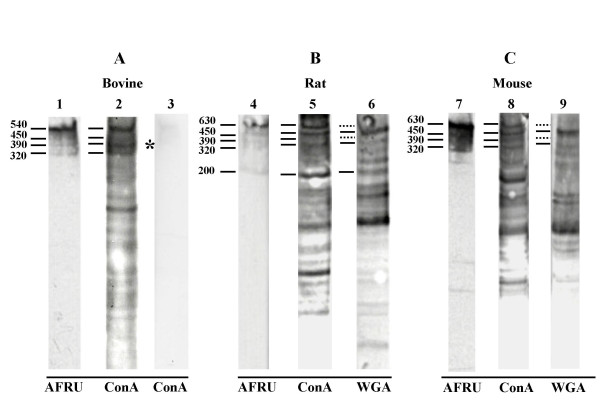
Immunoblots of SCO extracts of bovine (adult, lanes 1–3), rat (PN30, lanes 4–6) and mouse (PN30, lanes 7–9). Lanes 1, 4, 7: immunoblots using AFRU. Lanes 2, 5, 8: concanavalin A (Con A) binding. Lanes 6, 9: wheat germ agglutinin (WGA) binding. Lane 3: concanavalin A blocked with mannose prior to binding. No binding is detected (asterisk). Numbers: molecular weight in kDa. Dotted lines: compounds not binding WGA.

Proteins extracted from the rat, mouse and bovine SCO were blotted and run in parallel for immunoreaction with AFRU and Con A and WGA binding (Fig. 2B, 4; Table 1). In bovine SCO, four bands that immunoreacted with AFRU (540, 450, 390 and 320 kDa) bound Con A (Fig. 4A, Table 1). The five polypeptides detected with AFRU in the rat SCO (630, 450, 390, 320, 200 kDa) bound Con A, but only the compounds of 450, 320 and 200 kDa bound WGA (Fig. 4B, Table 1).

The four polypeptides detected with AFRU in the mouse SCO (630, 450, 390, 320 kDa) were Con A-positive (Fig. 4C); only the 450 and 320 kDa compounds bound WGA (Fig. 4C, Table 1).

## Discussion

### Identification of the precursor and processed forms of the glycoproteins secreted by the rat and mouse SCO

Reissner's fiber results from the assembly of large molecular weight proteins released by the SCO into the CSF circulating through the cerebral aqueduct. The major constituent protein of RF is SCO-spondin. The aminoacid sequence of bovine SCO-spondin has been fully established by Meiniel [21]. In the bovine SCO, three major compounds of 540, 450 and 320 kDa, reacting with an antiserum raised against the constitutive proteins of RF (AFRU), have been described [12]. Due to the lectin binding properties and the subcellular localization of these three compounds, Nualart *et al*. [25] have selected a 15-aminoacid sequence from a region of SCO-spondin that was used to raise antibodies in rabbits. This antibody (anti-P15) specifically reacts with the bovine and rat SCO and RF and can be regarded as a specific marker of SCO-spondin [25]. In immunoblots of bovine SCO, anti-P15 reacts with the 540 and 450 kDa bands, indicating that both bands correspond to SCO-spondin, with the former being the precursor and the latter a processed form. The molecular weight deduced from the aminoacid sequence of bovine SCO-spondin is 540 kDa [30]. The nature of the 320 kDa protein has yet to be investigated.

In the rat and mouse SCO, the 540 kDa compound was absent; instead an AFRU-immunoreactive compound of 630 kDa was seen. This compound has immunoreactive and lectin-binding properties similar to the bovine 540 kDa SCO-spondin, indicating that it corresponds to a murine SCO-spondin. The sequence of the mouse SCO-spondin contains 4981 aminoacids with a theoretical protein mass of 535 kDa. When compared to the bovine SCO-spondin there is a 66.8% homology [30].

Considering the large number of cryptic sites of N-glycosylaton present in the sequence of SCO-spondin, the larger molecular mass of rat and mouse SCO-spondin could be due to a higher degree of glycosylation.

According to the lectin binding properties of all AFRU-immunoreactive glycoproteins present in the rat and mouse SCO (Table 1), the 630 and 390 kDa compounds would correspond to precursor forms present in the rough endoplasmic reticulum of the SCO secretory cells. Indeed, they bound Con A, a lectin with affinity for mannose residues added to the nascent protein in the rough endoplasmic reticulum, but did not bind WGA, a lectin specific for two sugar residues (glucosamine, sialic acid) added to the glycoprotein by the Golgi apparatus [2,4]. At variance, the 450, 320 and 200 kDa proteins did bind WGA, indicating that they are post-Golgi compounds located, most likely, in the secretory granules. The presence of the 200 kDa glycoprotein in RF is strongly supportive of the suggestion that this compound corresponds to a processed form.

Out of the four compounds of large molecular weight revealed by AFRU in the rat SCO, only the 320 kDa reacted with anti-P15 (Table 3). Since this antibody is specific for SCO-spondin, it may be postulated that the 320 kDa protein corresponds to SCO-spondin. However, anti-P15 did not reveal the 630 kDa compound that most likely corresponds to the precursor of the rat SCO-spondin. A likely explanation for this result is that anti-P15, reacting with a 15-aminoacid sequence of a protein that consists of about 4,500 amino acids, might not have access to its epitopes when rat SCO-spondin is in its precursor form.

Out of the eight bands consistently revealed by AFRU in the embryonic CSF, only four of them (200, 180, 145, 63 kDa) reacted with anti-P15. This suggests that the latter compounds would be derived from the SCO-spondin precursor (630 kDa) and the AFRU-positive, anti-P15-negative compounds derived from the second putative precursor (390 kDa).

### Are CSF-soluble compounds secreted by the SCO intracellularly processed and released into CSF, or "released" from RF?

The 200 kDa appears as a key protein, since it was present in the SCO, RF and embryonic and juvenile CSF of rats, and immunoreacted with AFRU and anti-P15. This indicates that this protein is a processed form of SCO-spondin released by the SCO cells into the CSF. In the rat, the 200 kDa compound represented the major constituent protein of RF. A steady-state RF/CSF equilibrium for this protein might explain the permanent presence of this compound in both, RF and CSF. On the other hand, the presence of the 200 kDa protein in the embryonic CSF, before the first RF has formed, and in the juvenile cisternal CSF, a compartment where RF is absent, indicates that this protein is secreted as a CSF-soluble protein and remains soluble in the circulating CSF.

The 63 kDa protein detected in all CSF samples and in some samples of SCO and RF, might also be regarded as a processed form secreted by the SCO to form RF as well as to be CSF-soluble.

The two proteins of 180 and 164 kDa, found in the embryonic CSF but absent from the juvenile SCO, CSF and RF, would correspond to processed forms released by the SCO during late embryonic life. Since immunoblot analysis of the embryonic SCO has not been performed, the possibility that these compounds were derived from a source other than the SCO has to be considered. Indeed, from E15 to PN1 the rat floor plate, that extends along the ventral midline of the spinal cord and hindbrain, secretes proteins immunoreactive with AFRU [31]. Although floor plate explants release these proteins to culture medium [32], there is no evidence that they are actually released into the embryonic CSF.

There were AFRU and anti-P15 immunoreactive proteins present in the rat RF and/or juvenile CSF (120, 50, 25 kDa) that were absent from the SCO. Since these compounds were consistently detected in all blots, it seems unlikely they result from artefactual degradation of larger proteins during handling and processing of the samples. The possibility that these proteins result from a post-release cleavage of compounds secreted by the SCO into the CSF has to be considered and investigated. A similar phenomenon has been described in bovines, where several AFRU-immunoreactive polypeptides forming RF are not present in the SCO [11,12,33]. The possibility of a post-release processing of high molecular mass glycoproteins released into the CSF of chick embryos has been suggested [17]. In the bovine, the wall of the central canal, especially at the lumbo-sacral level, contains AFRU-reactive cells [34]. These cells have not been detected in the rat and mouse central canal. However, considering that in the present investigation, rat RF was extracted together with the wall of the central canal, the possibility should be considered that AFRU-immunoreactive proteins present in the central canal/RF extract but absent in the SCO extract, may have originated from cells lining the central canal.

### The CSF-soluble compounds secreted by the rat SCO circulate in the internal and external CSF compartments

After the intraventricular injection of an antibody against RF-proteins, such an antibody immunoreacts with the proteins newly released by the SCO, forming insoluble antigen-antibody complexes on the surface of this gland that were detected by immunocytochemistry using anti-IgG as primary antibody [35]. The same procedure has been used in the present investigation. AFRU and anti-P15 injected into the ventricular CSF formed insoluble antigen-antibody complexes exclusively located on the surface of the SCO, indicating that the rat SCO does secrete AFRU and anti-P15 reactive compounds into the third ventricle. Since AFRU and anti-P15 reactive compounds are also present in the cisternal CSF (see above), it may be concluded that the CSF-soluble proteins secreted by the SCO into the third ventricle circulate through the aqueduct of Sylvius, fourth ventricle and subarachnoid space.

### In the fetal CSF the number and concentration of SCO secretory products is higher than in the CSF of juvenile rats

The developing CNS of various vertebrate species has been investigated using methods to visualize RF-proteins. Two structures, the floor plate and the SCO, have been shown to share the property to synthesize RF-like material [36,37]. The use of anti-RF sera has confirmed that both structures secrete proteins reacting with these antibodies [31,37,38]. It has also been shown that both, the floor plate and SCO of bovine embryos, express the SCO-spondin gene and synthesize the 540 kDa protein [39]. The rat floor plate synthesizes AFRU-immunoreactive proteins from E15 to PN1, with the highest activity around E18 [31]. *In vitro *studies have shown that the bovine floor plate cells release AFRU-reactive proteins of 540 and 60 kDa into the culture medium [32]. The rat SCO starts to secrete AFRU-reactive material at E14; by E18 it is fully developed and displaying a high secretory activity [1]. Since RF starts to form at PN1, and a RF proper is first seen in the central canal at PN7, it has been assumed that the compounds secreted by the fetal SCO remain soluble in the CSF [1].

In the present investigation, eight AFRU-immunoreactive compounds were consistently found in all CSF samples collected from E18 to PN1. Only four of these compounds (200, 63, 50, 25 kDa) were also detected in the CSF of juvenile rats. It may be assumed that the source of these proteins is the SCO since in the juvenile rat this gland is the only brain structure secreting AFRU-reactive proteins. On the other hand, the four AFRU-immunoreactive proteins present only in the CSF collected from E18 to PN1 could be secreted by the SCO, the floor plate or by both glands. Five AFRU-immunoreactive proteins have been shown to occur in the CSF of chick embryos [17]. The authors obtained evidence to suggest that in chick embryos, these proteins may be derived from both the SCO and floor plate.

### Functional significance of the existence of CSF-soluble proteins secreted by the SCO

The embryonic CSF may be regarded as the main component of the milieu of stem cells and progenitor cells of the germinal zone [40]. In rat embryos, the flowing CSF is in permanent and direct contact with the ventricular and subventricular zones and, after E17, it reaches the subarachnoid space to bathe the marginal zone of the brain cortex [41]. The CSF is an efficient route to convey signals between different regions of the brain [41,42]. In the adult rat, tracers injected into the ventricles move freely into the brain parenchyma through the ependymal lining [43-45]; similarly, tracers injected into the subarachnoid space enter the brain parenchyma through the pial surface [44,46-48]. However, the functional relationship between the ventricular and subrachnoid compartments and the parenchyma of the embryonic brain is not clear. There is a significant body of evidence indicating that CSF carries vital signal molecules to the germinal epithelium of the developing brain cortex [41,49,50]. CSF from rat embryos, used as culture medium, has the capacity to sustain the proliferative activity of the embryonic cerebral cortex [51]. Rat CSF collected at E19 has the highest capacity to induce neuronal proliferation, compared to CSF obtained at other developmental periods, indicating that the CSF composition changes during development [51]. Rat CSF promotes the neuronal differentiation from neurospheres obtained from stem cells of the subventricular zone of the lateral ventricle [52]. Several factors known to participate in brain development have been detected in the embryonic CSF, namely, fibroblast growth factor (FGF2), epidermal growth factor (EGF), transforming growth factor TGF-b), neural growth factor (NGF), brain-derived growth factor (BDGF), transthyretin [53-62]. The source of most of these signals in not known.

The eight AFRU-immunoreactive compounds detected in the CSF of rat embryos (present report), especially those that also react with anti-P15 (a SCO-spondin marker), are good candidates to participate in brain development. This possibility is supported by previous observations. The immunological blockage of the SCO during development by maternal transfer of antibodies leads to brain abnormalities [63]. There is strong evidence indicating that SCO-spondin promotes neuronal growth and differentiation [64]. The fact that the eight AFRU-immunoreactive compounds have been detected in the cisterna magna CSF indicates they follow the flow of the subarachnoidal CSF and may gain access to the marginal zone of the developing brain cortex. Worth mentioning is the fact that F-spondin, secreted by the floor plate, SCO-spondin secreted by the SCO and reelin, secreted by the Cajal-Retzius cells of the developing cortex, display regions of similarities in their aminoacid sequences and all of them are glycoproteins secreted into the extracellular space [21,65].

The four AFRU-immunoreactive compounds that continue to be present in the CSF of juvenile rats may also be involved in neurogenesis, specially that occurring in the subventricular zone of the lateral ventricle. Transplantation of SCO explants to a lateral ventricle of juvenile rats stimulates neuroblast proliferation and migration [66].

## Conclusion

The present investigation has shown that (i) during late embryonic life, the SCO secretes compounds that remain soluble in the CSF and reach the subarachnoid space; (ii) during postnatal life, there is a reduction in the number and concentration of CSF-soluble proteins secreted by the SCO. The molecular structure and functional significance of these proteins remain to be elucidated. A glycoprotein of 200 kDa, found in all rat samples, may be a key compound for SCO function.

## Competing interests

The author(s) declare that they have no competing interests.

## Authors' contributions

KV and ER initiated and designed the study. KV, SR, CY and ER analysed and interpreted all the data. KV and CO collected data or carried out experiments for the study. KV and ER contributed to the preparation of the m.s. All authors have read and approved the final version of the manuscript.
